# Effect of Chlorhexidine on durability of two self-etch adhesive systems

**DOI:** 10.4317/jced.56873

**Published:** 2020-07-01

**Authors:** Haleh Kazemi-Yazdi, Mahdieh Saeed-Nezhad, Sadaf Rezaei

**Affiliations:** 1 DDS, MSc, Specialist in Operative Dentistry, Associate Professor, Operative Dentistry Department, Faculty of Dentistry, Tehran Medical Sciences, Islamic Azad University, Tehran, Iran; 2 DDS, Private Office, Tehran, Iran; 3 DDS, Postgraduate Student of Operative Dentistry, Operative Dentistry Department, Faculty of Dentistry, Tehran Medical Sciences, Islamic Azad University, Tehran, Iran

## Abstract

**Background:**

Despite of the rapid development in the field of dental adhesives, the issue of reduction in dentin bond durability has still not been resolved. The activity of dentinal endogenous enzymes such as MMPs is one of the most important causes of failure in resin composite restorations. The aim of this study was to investigate the influence of Chlorhexidine on micro-tensile bond strength of two types of commercially available self-etch adhesives.

**Material and Methods:**

Twenty four sound and freshly extracted molars were selected. Four standardized flat mid-coronal dentinal disks were prepared from each tooth. The specimens were randomly assigned to 6 groups (n=16). Groups A(control group) and B were treated with Clearfill SE Bond based on the manufacturer’s instructions. Groups C and D were treated with 2% Chlorhexidine 60 seconds before applying Clearfill SE Bond. Groups E and F were treated with Peak Universal Bond according to the manufacturer’s instructions. All groups were stored in distilled water in room temperature. Microtensile bond strength in groups A, C, and E were tested 24 hours after preparation, while microtensile bond strength in groups B, D, and F were tested after 3 months storage and 3000 thermal cycles(5-55 °C). Statistical analysis was performed with SPSS 20 and µTBS test results were analyzed using the Two-way ANOVA test.

**Results:**

µTBS was not significantly different between groups A, C, and E after 24 hours (*P*>0.5). There was no significant difference between groups B (Clearfill SE Bond + Aging) and D (Clearfill SE Bond + 2% CHX + Aging). The Peak Universal µTBS significantly decreased after the aging procedure (*P*<0.001).

**Conclusions:**

Based on the findings of this study, pretreatment with 2% CHX had no negative effect on the Clearfill SE Bond µTBS. However the µTBS of 0.2% CHX contained Peak Universal adhesive decreased significantly after aging.

** Key words:**Self-etch adhesives, Micro-tensile bond strength, chlorhexidine, bond durability.

## Introduction

Low long-term durability of dentin bondings is one of the major problems in adhesive dentistry. Studies have shown that bonding to dentin is much more difficult than bonding to enamel due to dentin’s complex structure, different percentage of organic and inorganic materials, and excess moisture (1). Dentin bondings are classified according to their adhesion techniques and effect on smear layer. Self-etch bondings are more widely used due to their convenience of application and maintenance of hybrid layer; however, their strength is decreased over time in oral conditions (2). Reduced strength of dentin bondings results in inadequate marginal seal, loss of restoration, hypersensitivity, secondary caries, and irreversible pulpitis (3).

In 2004, Pashely *et al.* reported the effect of MMPs on reducing dentin bond strength (4). MMPs are a group of Ca- and Zn-dependant endopeptidases which can degrade the extracellular matrix components. MMPs are essentially proenzymes activated by proteinases, chemicals, and low PH (5). Dentin contains MMPs 2, 8, 9, and 20 (6). MMPs 2 and 9, known as types of gelatinase and collagenase respectively, are active in the hybrid layer (7). MMPs are activated by self-etch or total-etch adhesives and their activity in the hybrid layer degrades type 1 collagen and reduces bond durability (5-7). Various methods have been used to increase dentin bond durability, among which MMP inhibitors have received particular attention. Different compounds such as tetracycline, galardin, glutaraldehyde, and chlorhexidine have been introduced as MMP inhibitors, among which chlorhexidine has been shown to be the most effective agent (8). Chlorhexidine has been used as a pre-adhesion antiseptic prior to its introduction as a MMP inhibitor; moreover, its MMP inhibitory effect has been reported in some of the recent studies (5). In contrast, a number of studies have shown the ineffectiveness of chlorhexidine or even its adverse effects on bond durability (9-11).

Factors affecting the time-dependant reduction of bond strength in self-etch adhesives have been investigated by many studies. However, the effect of MMP inhibitors on prevention of bond strength reduction over time is still subject of debate (12).

Given the inconsistencies and information gaps regarding the role of MMP inhibitors in durability of self-etch bondings, present study aimed to investigate the effect of chlorhexidine , either separately prior to Clearfil SE bond or in combination with the Peak Universal adhesive, on micro-tensile bond strength 24 hours after bonding and after aging.

## Material and Methods

**Figure 1:**
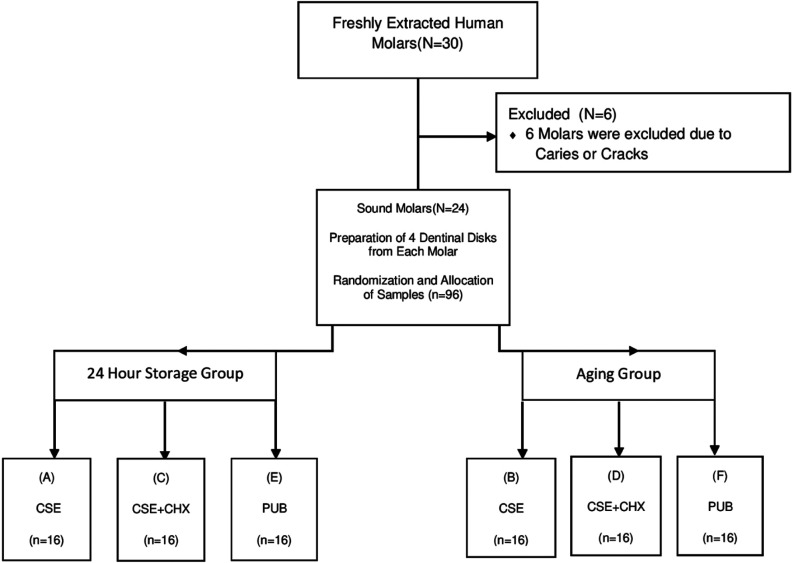
Consort Flow Diagram.

-Tooth Preparation and Sample Selection:

In this *in vitro* experimental study twenty-four freshly extracted caries-free human molars were collected from patients between 20-40 years old after obtaining the patient’s informed consent (Fig. 1). The teeth were disinfected in a 1% chloramine T solution for 1 week at 4°c , stored in distilled water, and used within 6 months after extraction (13).The sample size was calculated to be a minimum of 16 samples in each group based on the study by Deng *et al.*, using Minitab software. In the sample size calculation α=0.05, ß=0.2, and minimum significant difference and standard deviation were 6 and 3.1 respectively (14). For the purpose of the experiment, crowns were cut at the cementoenamel junction parallel to the occlusal surface using a low-speed diamond saw under water irrigation (Isomet; Buehler Ltd, Lake Bluff, IL, USA). Afterwards crowns were sectioned mesiodistally into buccal and lingual halves. The enamel on the buccal and lingual surfaces was ground with a long shank cylindrical bur and high speed handpiece in order to expose the dentin. Four dentinal disks were prepared to a thickness of 2 mm from each tooth. In order to standardize the smear layer, all specimens were grinded and polished with #400, #600, and #800 grit silicon carbide paper (waterproof silicon carbide paper, Matador, Germany) under running water for 30 seconds (11,12). The specimens were rinsed and randomly assigned to six groups(n=16).

Dentin Pretreatment and Adhesive Restorative Procedures:

The specifications and composition of the materials used in this study are summarized in Table 1.

**Table 1:**
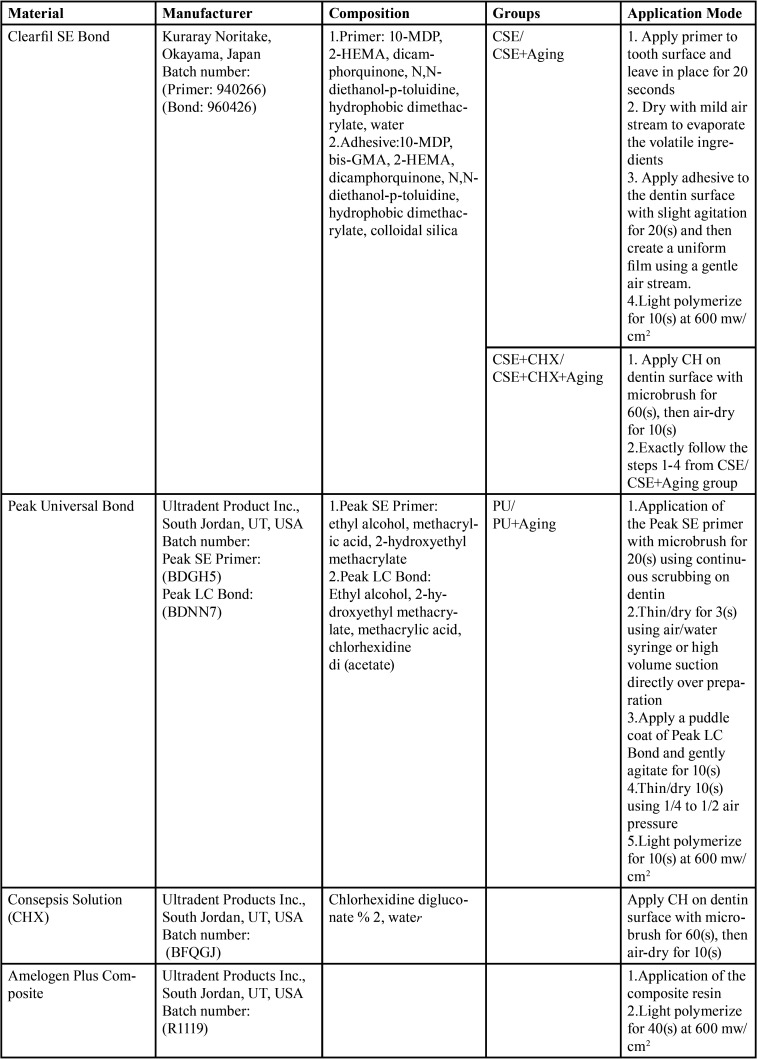
Composition, Application, and batch numbers of materials.

The examined groups were as following:

Group A (Control group): Clearfil SE Bond after 24 hour storage time(CSE)

Group B: Clearfil SE Bond after 3 months storage and 3000 thermal cycles (between 5-55° c with 20 seconds duel times) (CSE+Aging)

Group C: Clearfil SE Bond and %2 Chlorhexidine after 24 hour storage time (CSE+CHX)

Group D: Clearfil SE Bond and %2 Chlorhexidine after 3 months storage and 3000 thermal cycles (between 5-55° c with 20 seconds duel times) (CSE+CHX+Aging)

Group E: Peak Universal Bond after 24 hour storage time(PUB)

Group F: Peak Universal Bond after 3 months storage and 3000 thermal cycles(between 5-55° c with 20 seconds duel times ) (PUB+Aging)

Specimens were assigned to treatment groups and bonded according to manufacturer’s instructions. Two self-etch adhesives were used and investigated in this study. In groups A and B, the Clearfil SE Bond primer and bond (Kuraray, Okayama, Japan) were applied to dentin surfaces according to the manufacturer’s instructions and light polymerized for 10 seconds using a light curing unit (Demetron LC, Kerr Corp. , Orange County, CA, USA) with a light intensity of 600 mW/cm2.Whereas, in groups C and D, %2 CHX (Consepsis solution, Ultradent Products Inc. , South Jordan, UT, USA) was applied on the surfaces of dentinal disks for one minute prior to the application of the Clearfil SE Bond primer. In groups E and F, the Peak Universal SE primer and bond (Ultradent Product Inc. , South Jordan, UT, USA) were applied to dentin surfaces according to the manufacturer’s instructions.

After applying and light polymerization of bondings on dentinal disks, prefabricated plastic Tygon tubes with an internal diameter of 0.7 mm and height of 2 mm were placed on the bonded surfaces of specimens. An A2 shade of Amelogen Plus composite(Ultradent Product Inc., South Jordan, UT, USA) was packed into the Tygon tubes and light cured for 40 seconds with the light intensity of 600 mW/cm2. After the light polymerization, Tygon tubes were cut and separated from the composite cylinders using a scalpel. After removing the tubes, samples of groups A, C, and E were stored in distilled water for 24 hours at room temperature. Specimens of groups B, D, and F were stored in distilled water for 3 months at room temperature and then subjected to 3000 thermal cycles (between 5-55° c with 20 seconds duel times) (15,16).

Micro-tensile Bond Strength Testing:

The micro-tensile bond strength test was performed by a simplified universal testing machine (Bisco Inc., Schaumburg, IL, USA). Each sample was individually attached to the jig of the device using cyanoacrylate adhesive resin (Zapit, DVA, Corona, CA, USA). Load was applied at a crosshead speed of 1 mm/min and sustained to detachment of composite from the dentin surface. The failure load (recorded in N) was divided by the cross-sectional area of the bonding surface and the bond strength was calculated and reported in MPa.

Statistical Analysis:

Statistical analysis was performed with SPSS20(SPSS Inc., IL, USA) using two-way ANOVA test(Adhesive Type and Usage(CSE, CSE+CHX, PU) and Aging process(24 hours, 3 months storage+3000 thermal cycles)). The bond strength means and standard deviations(SD) for each group were tested for normal distribution using the Shapiro-Wilk test. Since the values were normally distributed across all groups, two-way ANOVA test was used for comparisons between groups. The significance threshold was set at *P*<0.05.

## Results

The mean values and standard deviations of the micro-tensile bond strengths are listed in Table 2. Statistical analysis revealed that µTBS of Peak Universal Bond (Adhesive with CHX in its composition) significantly decreased after the aging process (*P*<0.001). Analysis also showed that the groups used Peak Universal as the adhesive agent had significantly lower mean µTBS compared to groups which used Clearfil SE Bond after 3 months and 3000 thermal cycles (*P*<0.001).

**Table 2:**
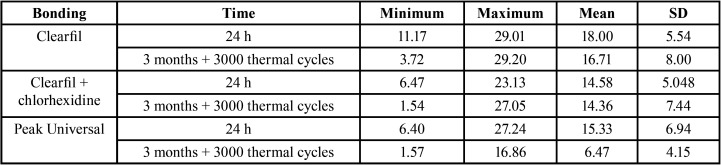
Minimum, maximum, mean, and standard deviation of micro-tensile bond strengths of speicmens according to the bonding type and the measurement time (Mpa).

The use of chlorhexidine to dentin prior to the application of Clearfil SE Bond had no significant effect on µTBS regardless of storage time (*P*>0.05).

In group A(CSE after 24 hours of storage), the mean µTBS of Clearfil SE Bond was 18 ± 5.54.In group B (CSE after aging process) in which samples’ adhesive procedure was the same as group A while the µTBS was measured after 3 months and 3000 thermal cycles, mean value was 16.71±8. Accordingly the bond strength of Clearfil SE Bond did not significantly decrease after the aging process (*P*>0.05).

Groups C and D were similar to groups A and B except CHX was applied before bonding procedure. The mean µTBS of group C (CSE+CHX after 24 hours) was 14.58±5.04 demonstrating no significant difference with group A(CSE after 24 hours). The mean µTBS of group D(CSE+CHX after aging process) was 14.36±7.44. There was no significant difference between groups C(CSE+CHX after 24 hours) and D(CSE+CHX after aging process) (*P*>0.05).

In group E(PU after 24 hours) the mean value of µTBS was 15.33±6.94. There was no significant difference between groups A, C, and E(CSE, CSE+CHX, PU after 24 hours of storage) (*P*>0.05). In group F(PU after aging procedure) the mean value of µTBS was 6.47±4.15 showing a significant reduction in µTBS compared to group E(PU after 24 hours of storage) (*P*<0.001).

Statistical analysis showed that µTBS of groups in which the bond strength was measured after only 24 hours of storage were not significantly different. However, after the aging process the reduction of µTBS of the Peak Universal bond was significantly more than the other two bonding procedures (*P*<0.001).

## Discussion

This study aimed to evaluate the effect of chlorhexidine either used separately before Clearfil SE Bond or used within the composition of Peak Universal Bond on µTBS of these self-etch adhesives, after 24 hours and after 3 months and 3000 thermal cycles.

 Based on the results and analysis of this study, the use of CHX, either in the composition of commercially available adhesive(Peak Universal Bond) or separately before Clearfil SE Bond, had no significant adverse effect on µTBS in the first 24 hours; however, µTBS of Peak Universal Bond which contains 0.2% CHX in its formulation decreased significantly after 3 months of storage in distilled water and 3000 thermal cycles.

Clearfil SE Bond is a mild self-etch adhesive which reacts with dentin through micromechanical and chemical mechanisms. Primary bonding is established through micromechanical mechanism whereas bond long term stability is associated with chemical mechanism. The stability of Clearfil SE Bond results from the presence of the mild acidic functional monomer, 10-MDP. Yoshida *et al.* have demonstrated that chemical interactions between MDP and hydroxyapatite creates a nano-layer at the adhesive interface, which increases the bond strength of the adhesive system. Additionally, the hydrophobic layer of adhesive prevents water penetration and deterioration of the bonding (17). All in all, Clearfil SE Bond has been considered in many studies as a golden standard of mild self-etch two-step adhesive systems (18). Therefore, in this study, Clearfil SE Bond was used in the control group.

Dentin matrix metalloproteinases(MMPs) are a family of proteolytic enzymes trapped within dentinal matrix, which have the ability to hydrolyze the organic matrix of demineralized dentin. The intrinsic MMPs can be activated by the acidic properties of adhesive systems. Etch & rinse and self-etching adhesives have been reported to have the ability to reactivate MMPs, causing the gradual degradation of bondings (5). On the one hand, it is well known that low concentrations of CHX can inhibit MMPs (19). There are some implications in the literature about the fact that CHX may increase the durability of bonding to dentin, especially in etch & rinse approach (Evidence of using CHX pretreatment in self-etch adhesives is still lacking since the effect of MMPs on the aging of self-etch bondings is controversial and debaTable (20)). On the other hand some researchers showed an unfavorable interaction between CHX and self-etching adhesives. Hypothetically, CHX reacts with dentin and the production of chlorine ions and crystal-shaped precipitates reduce the depth of etching, suggesting a chemical and physical interference (21). In addition, using CHX requires a separate step and in clinical situations it is not clear what kind or concentration of CHX should be used. Some manufacturers incorporate CHX in their adhesive systems in order to help clinicians avoid addition of one more step of separate CHX application during the bonding procedure. Adding CHX to commercially available adhesives may help CHX to being able to penetrate deeper into the adhesive zone. Due to increased depth of penetration, CHX will potentially be released slowly, therefore longer duration of antimicrobial and MMP inhibitory action could be possible (22).

Various studies reported a negative effect of CHX on the bond strength of adhesive systems (6). Campos *et al.* reported that dentin pretreatment with 2% CHX negatively affected µTBS of dentin substrates (23). In the current study, CHX had no significant adverse effect either at baseline or after aging on µTBS of Clearfil SE Bond. This result is consistent with those of Mobarak *et al*., Dalli *et al.*, and de Castro FL *et al.* who reported using CHX pretreatment on dentin do not interfere with the bond strength of Clearfil SE Bond adhesive system (10,24-25). Studies have shown that water molecules are required for activation of MMPs and hydrolysis of collagen peptide bonds. Since Clearfil SE Bond has a hydrophobic layer that prevents water penetration, it is most likely that the MMPs inhibitory effect of CHX in Clearfil SE Bond would not be significant (17).

Zhou *et al.* reported that CHX has a positive effect on µTBS of Clearfil SE Bond which is not in agreement with the results of current study. The reason for this difference may arise from the fact that in Zhou *et al.* study CHX was incorporated in the primer of Clearfil SE Bond (26).

The present study found that 0.2% CHX in the formulation of Peak Universal had no negative effect on baseline (24 hours) µTBS. C. Sabatini *et al.* also reported that when CHX is incorporated into a commercially available adhesive, no difference in bond strength was observed at baseline (9). Nishitani *et al.* reported that immediate µTBS of an experimental adhesive containing up to 1% CHX were not significantly different from CHX-free control adhesive (11). Miguel Angel Munoz *et al.* found out that Peak Universal adhesive, used in both total-etch and self-etch approach, showed mean µTBS statistically similar to those of the Clearfil SE Bond (27).

Based on the results of this research the Peak Universal bond strength significantly decreased after 3 months and 3000 thermal cycles. Miguel Angel Munoz *et al.* reported a significant decrease in Peak Universal µTBS after 6 months of water storage which is consistent with the findings of the present study. The reduced bond strength of the Peak Universal Bond after aging has been attributed to acidic PH of the primer, the absence of functional monomer for chemical bonding to the dentin, and the lack of a hydrophobic layer that prevents water penetration and bond degradation over time (28). According to the results of this work and previous studies, addition of CHX to the formulation of Peak Universal cannot necessarily prevent the reduction of its bond strength, as the bond strength of this product is not merely related to the activity of MMPs. The effect of CHX primarily depends on its concentration. Calcium ions, released from the dentin during the self-etch process, can essentially inhibit the effect of CHX on MMPs through their chelation property. Studies have shown that adding Ca to 0.03% CHX completely prevents its effect on MMPs (29). Therefore, CHX percentage in the primer or resin can significantly affect the resin- dentin bond stability. The low percentage of CHX may be completely neutralized by Ca released from the dentin. Chlorhexidine applied to the dentin is released over time with gradual decrease in its concentration, therefore the inhibitory effect of CHX on MMPs will decrease over time. The low concentration of CHX in the Peak Universal Adhesive cannot protect should be prevent the bond strength reduction over time. However, Maravic *et al.* reported that 0.2% CHX blended within Peak Universal adhesive monomer seems to increase µTBS of the adhesive at baseline and after 12 months storage in artificial saliva (30).

Despite extensive research on the mechanism of bonding deterioration, a definite conclusion has yet to be drawn and further investigation about the role of MMP inhibitors in enhancement of bonding durability especially in universal and self-etch adhesives is required.

## Conclusions

Within the limitations of this *in vitro* study, the results showed that chlorhexidine had no negative effect on the Clearfil SE Bond µTBS. However, the bond strength of the Peak Universal Bond containing 0.2% CHX, significantly decreased after the aging process.
